# Association Between Screening Practices and Other Risks and Breast Cancer Among Indonesian Women: A Case—Control Study

**DOI:** 10.3390/jcm14082699

**Published:** 2025-04-15

**Authors:** Primariadewi Rustamadji, Ratu Ayu Dewi Sartika, Pika Novriani Lubis, Edy Purwanto, Ismarulyusda Ishak, Amalia Ane Istamayu, Elvan Wiyarta

**Affiliations:** 1 Department of Anatomic Pathology, Faculty of Medicine, Universitas Indonesia, Jakarta 10430, Indonesia; primariadewi.rustamadji@ui.ac.id; 2 Department of Public Health Nutrition, Faculty of Public Health, Universitas Indonesia, Depok 16424, Indonesia; amalia.ane@ui.ac.id; 3 Department of Epidemiology, Faculty of Public Health, Universitas Indonesia, Depok 16424, Indonesia; pikanovr@gmail.com; 4 Department of Economics, Faculty of Economics and Business, Airlangga University, Surabaya 60286, Indonesia; edy.purwanto-2021@feb.unair.ac.id; 5 SurveyMETER, Yogyakarta 55282, Indonesia; 6 Program Sains Bioperubatan, Fakulti Sains Kesihatan Perubatan, Universiti Kebangsaan Malaysia, Jalan Raja Muda Abd Aziz, Kuala Lumpur 50300, Malaysia; ismarul@ukm.edu.my; 7 Intensive Care Department, University of Indonesia Hospital, Depok 16424, Indonesia; elvan.wiyarta@ui.ac.id; 8 Service Department, Risetku, South Jakarta 12820, Indonesia

**Keywords:** breast cancer, screening, mammogram, urban, rural

## Abstract

Breast cancer is the predominant cause of cancer in developing nations, and screening through breast self-examinations and mammograms is crucial in mitigating morbidity and mortality. Nonetheless, geographic disparities in screening methods persist, attributable to sociodemographic variation and healthcare accessibility. **Background/Objectives:** This study aimed to analyze the influence of women’s screening practices for breast cancer and other risks, stratified by urban and rural areas in Indonesia. **Methods:** A case–control design was adopted, including all women who had breast cancer in 2014 as the study subjects. The Indonesian Family Life Survey data from 2007, with subjects aged at least 15 years, and from 2014 were used. Unconditional logistic regression was used to analyze the risk factors of breast cancer. **Results:** After controlling for confounders, the odds of breast cancer diagnosis were higher in women who performed breast self-examination (BSE) (aOR 10.22; 95% CI 1.04–50.81 and aOR 11.10; 95% CI 3.32–37.08) and those married before the age of 19 (aOR 4.81; 95% CI 1.93–6.05 and aOR 5.35; 95% CI 1.49–19.7), in urban and rural areas, respectively. In addition, women who had undergone mammography (aOR 48.04; 95% CI 10.33–83.45) had significantly higher odds of being diagnosed with breast cancer in urban areas. In rural areas, a paternal history of cancer-related death had higher odds of breast cancer (aOR 30.63; 95% CI 6.04–60.41) than those without a parental history of cancer. **Conclusions:** This study highlights the importance of intensifying national breast cancer screening, including BSE campaigns and expanding mammography infrastructure, particularly in rural areas, for improving breast cancer prevention and early diagnosis.

## 1. Introduction

The battle against cancer has emerged as a major global public health issue. The International Agency for Research on Cancer (IARC) reported 20 million new cases of cancer and 10 million deaths by 2022, with the number projected to increase to 35 million by 2050 [1,2]. The overall cancer burden continues to rise in low- and middle-income countries (LMICs), particularly in Asia [3]. In Southeast Asia, breast cancer remains the most diagnosed cancer and the leading cause of cancer-related death in Indonesia, with 66,300 new cases and 22,600 deaths reported in 2022 [4,5]. This concerning observation is reflected in adjacent countries, including the Philippines (33,100 cases and 11,900 deaths), Vietnam (24,600 cases and 10,000 deaths), Thailand (21,600 cases and 7600 deaths), and Malaysia (8400 cases and 3500 deaths). These alarming statistics emphasize the urgent need for enhanced awareness, early detection, and access to effective treatment options in this region [4,5].

Notwithstanding global progress in cancer prevention and treatment, most breast cancers are diagnosed late (at an advanced stage), where curative treatment is no longer possible in most cases [6]. Screening practices through breast self-examination (BSE) and mammography are critical to reducing morbidity and enhancing survival rates effectively [7,8]. However, participation in screening programs remains low in many LMICs, including Indonesia [6]. This low participation is influenced by various factors, such as sociodemographic variables, healthcare accessibility, and cultural perceptions towards cancer screening [9,10].

The risk factors for breast cancer are multifactorial, encompassing genetic predisposition, hormonal influences, sociodemographic characteristics, and lifestyle. A family history of breast cancer increases risk significantly, with studies reporting a two-fold higher risk among individuals with affected first- or second-degree relatives. Hormonal factors, such as age at menarche, parity, breastfeeding history, menopausal status, and contraceptive use, have been linked to breast cancer [11,12]. For instance, pre-menopausal women exhibited a 40% higher risk compared to post-menopausal women [13]. In addition, unhealthy behaviors such as smoking, dietary choices, physical inactivity, high-calorie diets, and obesity further exacerbate cancer risk [11,12,14]. Nevertheless, these lifestyles can be modified, emphasizing the necessity of early screening to ensure timely prevention before the age of pre-menopause [15].

Geographic disparities further compound the challenges in addressing breast cancer prevention and management in Indonesia. Screening rates are generally lower in rural areas compared to urban areas, primarily due to barriers such as inadequate healthcare facilities, financial constraints, and lower levels of education. Rural populations are also characterized by older age, lower socioeconomic status, and greater frequency of unhealthy behaviors, including smoking and infrequent physical activity, which contribute to a higher incidence of cases and late-stage diagnoses [8,11,14,16,17]. In contrast, urban populations, while benefiting from better healthcare access, encounter additional risk factors, including postponed childbirth, diminished parity, and heightened intake of high-fat diets [11]. These contrasting patterns underscore the complexity of breast cancer prevention in diverse populations. However, studies on the geographic factors in breast cancer cases within LMICs, exclusively in Asia, are insufficient. Comprehending the risk factors affecting breast cancer is essential for formulating tailored public health interventions that address the unique challenges faced by each demographic group. Therefore, this study aims to examine the influence of screening practices and other risks on breast cancer cases, differentiated by urban and rural settings.

## 2. Materials and Methods

This study utilized secondary data from the longitudinal study of the Indonesian Family Life Survey (IFLS), specifically from the waves in 2007 and the latest data from 2014. IFLS data are the only cohort data in Indonesia that comprehensively describe the population. The data included 7224 households selected from 13 out of 26 provinces in Indonesia, representing approximately 83% of the national population and capturing its diversity [18].

A community-based unmatched case–control study design was employed, considering the relatively low prevalence of cancer in Indonesia, estimated at 1.8% [19], and the nature of the long latency of cancer development [11]. The eligible cases were women diagnosed with breast cancer in 2014. Meanwhile, the controls were women without a breast cancer diagnosis in the same year. We excluded the missing data caused by technical issues, refusal, or ignorance of respondents. The detailed explanations are available at the IFLS website (https://sites.rand.org/labor/family/software_and_data/FLS/IFLS/IFLS2/doc/volume2.zip, accessed on 5 September 2024). To investigate disparities in breast cancer risk factors, participants were stratified based on urban and rural residence, as classified by the Indonesian Bureau of Statistics (BPS) at the time of the survey interview.

The dependent variable was self-reported breast cancer diagnosed by a doctor in 2014, while the independent variables included various risk factors of breast cancer, such as screening practices, individual characteristics, behavioral factors, and enhancing and supporting factors. Cancer screening practices were assessed using two questions: “Have you heard about mammograms before?” and “How many times have you had a breast self-examination/mammogram in the past 12 months?”.

Individual characteristics included age, education, socioeconomic status, occupation, age at menarche, marital status, age at marriage, number of live births, stillbirth, abortion, miscarriage, and menopause status. Women who married before the age of 19 years were classified as having an early marriage based on Indonesia’s legal age definition.

Behavioral factors included breastfeeding, smoking status, risky eating behavior, and contraception use. Respondents were asked whether they had a smoking habit. Meanwhile, enhancing factors included obesity status (body mass index) and parental history of cancer-related deaths. The supporting factors, including health insurance ownership and accessibility to the nearest healthcare centers (measured in terms of distance, travel time, and cost), were also evaluated. The mean values were used to determine the cut-off for categorizing the latter variables.

We used IBM SPSS Statistics ver. 29.0 software (IBM Co., Armonk, NY, USA, RRID: SCR_002865) for all analyses. Descriptive data are represented as means and standard deviations (SDs). Unconditional logistic regression was performed for bivariate and multivariate analysis to determine the odds ratio (OR) for each potential breast cancer risk factor. Variables with a *p*-value < 0.25 in bivariate analysis were considered potential confounders and were included in the final model to calculate the adjusted odds ratio (aOR). Statistical significance was determined at *p*-value < 0.05, while *p*-values < 0.10 indicated a tendency to associate. All analyses were performed without weighting, as each data unit was assigned equal influence. The study adhered to the Strengthening the Reporting of Observational Studies in Epidemiology (STROBE) guidelines to ensure rigorous reporting.

## 3. Results

### 3.1. Descriptive Characteristics

**Figure 1:**
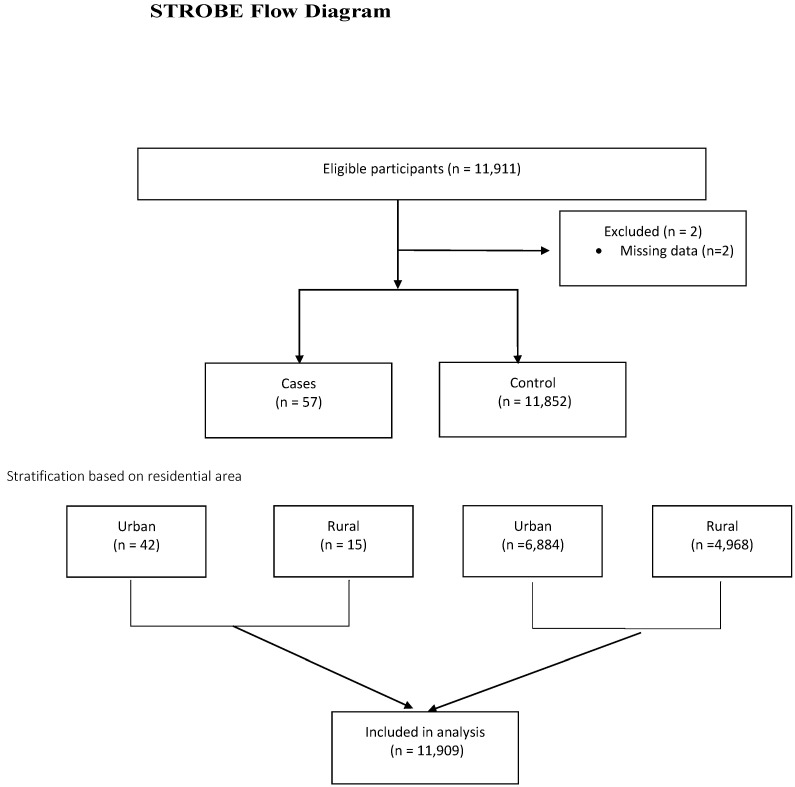
STROBE flow diagram of case–control studies.

A total of 11,911 respondents, comprising 57 cases and 11,852 controls, were included in this study, with 6926 and 4983 in urban and rural areas, respectively. Both cases and controls were selected from the same database to minimize selection bias. Due to the negligible number of missing data (<1%), we omitted the missing data from our analysis (Figure 1). Table 1 shows that urban respondents had a lower average age and age at menarche than rural respondents (35.9 vs. 36.9 years and 13.9 vs. 14.1 years, respectively). Meanwhile, the average age at marriage and menopause was higher in urban areas (22.1 vs. 20.9 years and 47.2 vs. 46.5 years).

### 3.2. Bivariate Analysis

Table 2 shows the odds of breast cancer diagnosis based on various factors. In urban areas, women who performed BSE had 4 times higher odds of a diagnosis of breast cancer (OR 3.6; 95% CI 1.92–6.76), and those aware of mammograms had 5 times higher odds (OR 5.3; 95% CI 2.76–10.12). Furthermore, there is an 8-fold increase in odds for those who carried out a mammogram in the past year (OR 40.9; 95% CI 12.14–137.87). Additional risk factors included marriage before age 19 years (OR 2.5; 95% CI 1.14–5.63), health insurance ownership (OR 1.8; 95% CI 0.95–3.29), education level of at least in high school (OR 2.0; 95% CI 1.09–3.82), and lower economic status (quintile 1–3) with OR 3.2; 95% CI 1.15–8.72, OR 3.3; 95% CI 1.21–9.23, and OR 2.8; 95% CI 1.09–7.28, respectively. Meanwhile, in rural areas, the odds of being diagnosed with breast cancer were 10 times higher for women who had performed BSE (OR 9.9; 95% CI 3.46–28.42) and 6 times higher for those who heard of mammograms (OR 5.7; 95% CI 1.59–20.35). Other significant risk factors included early marriage (OR 2.7; 95% CI 0.89–8.38), a history of paternal cancer-related death (OR 19.9; 95% CI 4.35–91.47), and lower economic status (quintiles 2 and 3) (OR 10.7; 95% CI 1.19–96.39, and OR 8.2; 95% CI 0.92–73.97).

### 3.3. Multivariate Analysis

The multivariate analysis showed that in urban areas, women who had undergone a mammogram in the past 12 months had significantly higher odds of being diagnosed with breast cancer (aOR 48; 95% CI 10.33–83.45). Additionally, women who had performed BSE had 10 times higher odds (aOR 10.22; 95% CI 1.04–50.81) and 5 times higher odds if the age at marriage was below 19 years (aOR 4.8; 95% CI 1.93–6.05). In rural areas, the odds of breast cancer were 11 times higher among women who had performed BSE (aOR 11.1; 95% CI 3.32–37.08) and greater in those who aged below 19 years at marriage (adOR 5.3; 95% CI 1.49–19.08), while those with a paternal history of cancer-related death had 19.4 times higher odds (aOR 19.4; 95% CI 3.75–100.78) (Table 3).

## 4. Discussion

This study is the first to associate the breast cancer risk factors, stratified by urban and rural areas in Indonesia, using secondary data from the longitudinal Indonesian Family Life Survey (IFLS). The results confirmed that higher screening participation is strongly associated with breast cancer diagnosis in both settings. Despite the critical role of screening in breast cancer management, preventive initiatives in Indonesia remain underpromoted and underutilized [20]. Similar patterns of low screening participation have been observed in other LMICs, including countries in Africa and India [21,22]. The underlying causes may include limited patient preference, insufficient physician recommendations, and systemic barriers to healthcare access [8,9].

Our findings indicated that women who participate in screening are more likely to be diagnosed with breast cancer. This conclusion is corroborated by a previous study mentioning that increased screening efforts aligned with the rising incidence of breast cancer. Early diagnosis of breast cancer facilitates timely treatment, leading to higher survival rates and lower mortality rates [3]. While mammography, a gold standard breast cancer screening [3], is occasionally criticized for false positives, false negatives, and overdiagnosis [23], its role in reducing breast cancer death is well-documented [24]. Another screening for breast cancer, BSE, continues to be promoted as an accessible screening method in Indonesia despite ongoing debates regarding its effectiveness [20,25]. While less accurate than mammograms, BSE is feasible, economical, and serves as an option for women under 40 years or those without access to mammography. Evidence suggests that BSE can aid in early tumor detection and improve survival outcomes [26,27,28]. A randomized controlled trial in Iran concluded that the women trained in regular BSE were diagnosed at earlier stages than the control group, thereby reducing breast cancer mortality [29]. However, limited knowledge about BSE techniques and timing remains a challenge, with many women unsure whether they are performing the procedure correctly [21,30]. Furthermore, intensifying BSE campaigns and incorporating mammography with well-trained healthcare personnel should be considered to broaden screening coverage.

This study also highlights significant geographic disparities in screening participation influenced by socioeconomic and healthcare-related factors. Women in rural areas face additional barriers, including limited healthcare facilities, insufficient physician advocacy, and fewer mammogram devices, as reported in previous studies in the United States and China [31]. Currently, Indonesia has only 43 mammograms registered in clinics and hospitals nationwide, with only four actively providing services [32]. These resources are disproportionately concentrated only in urban areas, further marginalizing rural populations. Additionally, economic constraints and lack of health insurance significantly influence participation in mammogram screening [11]. However, our results indicate that women having health insurance have a greater chance of diagnosing breast cancer (OR 1.78; 90% CI 0.954; 3.287) than uninsured women. It contradicts a report from other LMICs, where limited insurance coverage contributes to lower screening rates [28]. This discrepancy may be due to the fact that we do not distinguish whether the screening was self-initiated or based on a doctor’s recommendation. Individuals diagnosed with breast cancer may often receive a physician’s suggestion for routine mammography before the diagnosis. Although the implementation of Universal Health Coverage via the National Health Insurance program was established in Indonesia over one decade ago, mammography is recommended primarily for symptomatic cases [20]. To improve early detection, we recommend expanding resources to enhance mammogram accessibility, particularly in rural areas.

Socioeconomic conditions are critical determinants of screening participation and the occurrence of breast cancer and are correlated with the determination of marriage age. The percentage of early marriage, one of the risks of breast cancer in Indonesia, is relatively high at 11%. Delaying marriage may impose additional social and financial burdens on the family [33]. Our result stated that women who marry at a younger age had an increased chance of developing breast cancer. Previous studies suggest that the risk of developing breast cancer decreases by 17% for each additional year of delayed marriage [34,35].

Beyond systemic and socioeconomic barriers, cultural and personal factors also play a role. In Indonesia, breast cancer screening remains taboo for many women due to embarrassment or sensitivity regarding body exposure. In some cases, women must obtain their husband’s permission for check-ups at health facilities. Fear of pain during mammograms, apprehension about test results, and lack of awareness regarding the benefits of routine screening further discourage participation [28,30,36]. To increase screening practices, effective public health campaigns focusing on the causes, risks, and symptoms of breast cancer are essential to address these challenges [21]. Health promotion efforts should leverage diverse communication channels, including mass media and community-based interventions, to enhance awareness and engagement [37].

We also suggested the potential influence of genetics on breast cancer risk. A strong family history, particularly among first-degree relatives, significantly increases the risk [38]. In our study, the analysis reported that a history of paternal death due to cancer is associated with breast cancer risk, both before and after controlling for confounders, particularly in rural populations. While the underlying mechanism remains unclear, previous studies found that the history of paternal cancer was associated with an increased incidence of lobular breast cancer in descendants (OR 1.9; 95% CI 1.2–2.8), potentially due to genetic alleles in the father that predispose daughters to breast cancer [39].

A large sample size with very few missing datasets is an advantage of this study. However, limitations include potential recall bias from self-reported data and differences in case–control sample sizes when stratified by residence. Social desirability bias may result in overreporting data in some responses, although this is unlikely to have differentially affected the groups [40]. Furthermore, our study could not eliminate screening bias; women diagnosed with cancer may experience increased screening frequency due to symptoms, genetic predispositions, or physician recommendations.

## 5. Conclusions

In conclusion, the results of this study provide strong evidence for initiating a nationwide breast cancer screening program. Intensifying the BSE campaign and enhancing mammography infrastructure and resources, especially in rural areas, will facilitate seamless implementation and enhance early detection efforts. Further in-depth studies with larger sample sizes are necessary to explore the underlying reasons for the low uptake of screening practices and to develop targeted interventions for improving breast cancer prevention and early diagnosis in Indonesia.

## Figures and Tables

**Table 1:** Characteristic of respondents.

Variables	Urban (n = 6926)	Rural (n = 4983)
	Mean ± SD (Min–Max)	Mean ± SD (Min–Max)
Age (2007; years)	35.86 ± 14.11 (15–94)	36.92 ± 15.13 (15–88)
Age at menarche (years)	13.88 ± 1.61 (12–41)	14.05 ± 1.77 (12–64)
Age at marriage (years)	22.08 ± 5.45 (15–70)	20.97 ± 5.46 (15–55)
Menopausal age (years)	47.17 ± 4.47 (25–58)	46.45 ± 4.81 (27–57)

**Table 2:** Association between screening practices, individual and sociodemographic characteristics, and breast cancer in urban and rural areas.

Risk Factors	Urban (n = 6926)				Rural (n = 4983)			
	Case	Control			Case	Control		
	n (%)	n (%)	*p*-Value	OR (95% CI)	n (%)	n (%)	*p*-Value	OR (95% CI)
Breast self-examination								
Ever	19 (48.7)	1375 (20.9)	0.000 *	3.6 (1.916; 6.763)	7 (50.0)	429 (9.2)	0.000 *	9.923 (3.464; 28.423)
Never	20 (51.3)	5210 (79.1)			7 (50.0)	4257 (90.8)		
Have heard about mammograms								
Yes	14 (35.0)	611 (9.2)	0.000 *	5.304 (2.755; 10.120)	3 (20.0)	198 (4.2)	0.024 *	5.697 (1.595; 20.350)
No	26 (65.0)	6018 (90.8)			12 (80.0)	4512 (95.8)		
Had a mammogram test in the last 12 months								
Perform	6 (42.9)	11 (1.8)	0.000 *	40.909 (12.139; 137.866)	0 (0.0)	2 (1.0)	1.000	NA
Not perform	8 (57.1)	600 (98.2)			3 (100)	196 (99.0)		
Have health insurance								
Yes	17 (40.5)	1910 (27.7)	0.096 ^+^	1.771 (0.954; 3.287)	5 (33.3)	1009 (20.3)	0.206	1.962 (0.669; 5.752)
No	25 (59.5)	4974 (72.3)			10 (66.7)	3959 (79.7)		
Age								
≥40 years	12 (28.6)	2445 (35.5)	0.438	0.726 (0.371; 1.421)	7 (46.7)	1908 (38.4)	0.696	1.403 (0.508; 3.876)
<40 years	30 (71.4)	4439 (64.5)			8 (53.3)	3060 (61.6)		
Age at menarche								
≤14 years	19 (61.3)	2312 (45.5)	0.114	1.898 (0.920; 3.919)	5 (38.5)	1509 (40.1)	1.000	0.934 (0.305; 2.859)
>14 years	12 (38.7)	2772 (54.5)			8 (61.5)	2254 (59.9)		
Age at marriage								
<19 years	25 (75.8)	3089 (55.2)	0.018 *	2.536 (1.142; 5.633)	8 (61.5)	1564 (36.9)	0.084 ^+^	2.738 (0.894; 8.383)
≥19 years	8 (24.2)	2507 (44.8)			5 (38.5)	2676 (63.1)		
Marital status								
Not married	4 (9.5)	448 (6.5)	0.351	1.512 (0.537; 4.256)	1 (6.7)	169 (3.4)	0.406	2.028 (0.265; 15.514)
Married	38 (90.5)	6436 (93.5)			14 (93.3)	4799 (96.6)		
Occupation status								
Unemployment	20 (47.6)	2565 (37.3)	0.221	1.531 (0.834; 2.810)	3 (20.0)	1592 (32.0)	0.413	0.530 (0.149; 1.881)
Employment	22 (52.4)	4319 (62.7)			12 (80.0)	3376 (68.0)		
Education level								
High school or higher education	26 (61.9)	3047 (44.3)	0.032 *	2.046 (1.096; 3.821)	6 (40.0)	1087 (21.9)	0.113	2.380 (0.845; 6.702)
Less than high school	16 (38.1)	3837 (55.7)			9 (60.0)	3881 (78.1)		
Tobacco smoking								
Yes	1 (2.4)	169 (2.5)	1.000	0.969 (0.133; 7.087)	1 (6.7)	199 (4.0)	0.460	1.712 (0.224; 13.082)
No	41 (97.6)	6715 (97.5)			14 (93.3)	4769 (96.0)		
Body mass index								
>25 kg/m^2^	15 (35.7)	3301 (48.0)	0.151	0.602 (0.320; 1.133)	6 (40.0)	1947 (39.2)	1.000	1.033 (0.367; 2.907)
<25 kg/m^2^	27 (64.3)	3575 (52.0)			9 (60.0)	3017 (60.8)		
Number of births								
≥1 child	13 (31.7)	2956 (44.3)	0.145	0.585 (0.302; 1.131)	8 (53.3)	2252 (47.4)	0.839	1.270 (0.460; 3.507)
No child	28 (68.3)	3722 (55.7)			7 (46.7)	2502 (52.6)		
History of breastfeeding								
Never	0 (0)	82 (4.6)	1.000	NA	0 (0.0)	76 (6.0)	1.000	NA
Ever	10 (100)	1684 (95.4)			4 (100)	1183 (94.0)		
History of stillbirth								
Yes	0 (0.0)	87 (1.3)	1.000	NA	0 (0.0)	85 (1.8)	1.000	NA
No	41 (100)	6591 (98.7)			15 (100)	4669 (98.2)		
History of miscarriage								
Yes	2 (4.9)	444 (6.6)	1.000	0.720 (0.173; 2.992)	1 (6.7)	302 (6.4)	1.000	1.053 (0.138; 8.034)
No	39 (95.1)	6234 (93.4)			14 (93.3)	4452 (93.6)		
History of abortion								
Ever	0 (0.0)	47 (1.1)	1.000	NA	0 (0.0)	18 (0.6)	1.000	NA
Never	28 (100)	4344 (98.9)			8 (100)	3140 (99.4)		
Menopausal status								
Yes	1 (2.4)	136 (2.0)	0.569	1.210 (0.165; 8.862)	1 (6.7)	98 (2.0)	0.260	3.550 (0.462; 27.260)
No	41 (97.6)	6748 (98.0)			14 (93.3)	4870 (98.0)		
Oral contraception								
Ever	15 (53.6)	2241 (51.0)	0.938	1.107 (0.526; 2.332)	4 (50.0)	1474 (46.7)	1.000	1.142 (0.285; 4.576)
Never	13 (46.4)	2150 (49.0)			4 (50.0)	1684 (53.3)		
Injection contraception								
Ever	18 (64.3)	3187 (72.6)	0.443	0.680 (0.313; 1.477)	4 (50.0)	2402 (76.1)	1.000	0.315 (0.079; 1.261)
Never	10 (35.7)	1204 (27.4)			4 (50.0)	756 (23.9)		
Implant contraception								
Ever	1 (3.6)	292 (6.6)	1.000	0.520 (0.070; 3.840)	0 (0.0)	399 (12.6)	0.607	NA
Never	27 (96.4)	4099 (93.4)			8 (100)	2759 (87.4)		
Father died from cancer								
Yes	1 (2.4)	97 (1.4)	0.451	1.707 (0.232; 12.532)	2 (13.3)	38 (0.8)	0.006 *	19.955 (4.353; 91.472)
No	41 (97.6)	6787 (98.6)			13 (86.7)	4929 (99.2)		
Mother died from cancer								
Yes	1 (2.4)	96 (1.4)	0.448	1.725 (0.235; 12.666)	0 (0)	61 (1.2)	1.000	NA
No	41 (97.6)	6788 (98.6)			15 (100)	4906 (98.8)		
Healthcare accessibility (distance)								
>5 km	3 (18.8)	300 (21.5)	1.000	0.843 (0.239; 2.978)	3 (75.0)	414 (48.1)	0.357	3.239 (0.336; 31.263)
≤5 km	13 (81.3)	1096 (78.5)			1 (25.0)	447 (51.9)		
Healthcare accessibility (time)								
>10 min	6 (54.5)	526 (40.0)	0.364	1.802 (0.547; 5.936)	2 (50.0)	447 (48.4)	1.000	1.065 (0.149; 7.592)
≤10 min	5 (45.5)	790 (60.0)			2 (50.0)	476 (51.6)		
Healthcare accessibility (price)								
IDR > 16,500	0 (0.0)	156 (10.7)	0.392	NA	3 (75.0)	444 (49.0)	0.366	3.122 (0.323; 30.122)
IDR ≤ 16,500	15 (100)	1301 (89.3)			1 (25.0)	462 (51.0)		
Socioeconomic status (monthly household expenditure)								
Quintile 1	5 (11.9)	1342 (19.5)	0.026 *	3.160 (1.145; 8.720)	5 (33.3)	1576 (31.7)	0.133	2.747 (0.735; 10.271)
Quintile 2	5 (11.9)	1420 (20.6)	0.020 *	3.344 (1.212; 9.226)	1 (6.7)	1233 (24.8)	0.034 *	10.745 (1.198; 96.387)
Quintile 3	6 (14.3)	1436 (20.9)	0.033 *	2.818 (1.090; 7.284)	1 (6.7)	946 (19.0)	0.060 ^+^	8.244 (0.919; 73.968)
Quintile 4	11 (26.2)	1411 (20.5)	0.301	1.510 (0.691; 3.300)	4 (26.7)	754 (15.2)	0.484	1.643 (0.409; 6.600)
Quintile 5	15 (35.7)	1274 (18.5)			4 (26.7)	459 (9.2)		

* *p*-value < 0.05; + *p*-value < 0.10.

**Table 3:** Final multivariable determinants of breast cancer in urban and rural areas.

Variables	Breast Cancer		
	*p*-Value	aOR	95% CI
Urban areas ^a^			
Had a mammogram test in the last 12 months			
Not perform	Ref	1	
Perform	0.000 *	48.038	10.327; 83.453
Breast self-examination			
Never	Ref	1	
Ever	0.046 *	10.223	1.037; 50.809
Age at marriage			
≥19 years	Ref	1	
<19 years	0.052 ^+^	4.814	1.925; 6.049
Rural areas ^b^			
Breast self-examination			
Never	Ref	1	
Ever	0.000 *	11.102	3.324; 37.077
Age at marriage			
≥19 years	Ref	1	
<19 years	0.010 *	5.345	1.498; 19.071
Father died from cancer			
No	Ref	1	
Yes	0.000 *	30.632	6.038; 60.406

aOR, adjusted odds ratio; CI, confidence interval. ^a^ Initial model includes breast self-examination, heard about the mammogram, mammogram test, age at menarche, age at marriage, number of births, ownership of insurance, education level, occupation status, socioeconomic status, and body mass index. ^b^ Initial model includes breast self-examination, heard about the mammogram, age at marriage, ownership of insurance, education level, meat consumption, socioeconomic status, and father died from cancer. * *p*-value < 0.05; + *p*-value < 0.10.

## Data Availability

The datasets are available on the RAND Corporation’s website, as the institute that conducted the survey and owns the IFLS data: https://www.rand.org/well-being/social-and-behavioral-policy/data/FLS/IFLS.html, accessed on 5 September 2024.

## Abbreviations

The following abbreviations are used in this manuscript:

IARC	International Agency for Research on Center
IFLS	Indonesia Family Life Survey
BSE	Breast Self-Examination
LMIC	Low- and Middle-Income Countries
aOR	Adjusted Odds Ratio
CI	Confidence Interval
